# Cerium Oxide Nanoparticles Rescue α-Synuclein-Induced Toxicity in a Yeast Model of Parkinson’s Disease

**DOI:** 10.3390/nano10020235

**Published:** 2020-01-29

**Authors:** Roberta Ruotolo, Giuseppe De Giorgio, Ilaria Minato, Massimiliano G. Bianchi, Ovidio Bussolati, Nelson Marmiroli

**Affiliations:** 1 Department of Chemistry, Life Sciences and Environmental Sustainability, University of Parma, 43124 Parma, Italy; giuseppe.degiorgio@unipr.it (G.D.G.); ilaria.minato@studenti.unipr.it (I.M.); 2 Department of Medicine and Surgery, University of Parma, 43125 Parma, Italy; massimiliano.bianchi@unipr.it (M.G.B.); ovidio.bussolati@unipr.it (O.B.); 3 The Italian National Interuniversity Consortium for Environmental Sciences (CINSA), 43124 Parma, Italy

**Keywords:** cerium oxide nanoparticles, nanoceria, Parkinson’s disease, α-synuclein, yeast model, amyloid aggregates, oligomer detoxification, cluster of lipid vesicles, neurodegenerative disease

## Abstract

Over the last decades, cerium oxide nanoparticles (CeO_2_ NPs) have gained great interest due to their potential applications, mainly in the fields of agriculture and biomedicine. Promising effects of CeO_2_ NPs are recently shown in some neurodegenerative diseases, but the mechanism of action of these NPs in Parkinson’s disease (PD) remains to be investigated. This issue is addressed in the present study by using a yeast model based on the heterologous expression of the human α-synuclein (α-syn), the major component of Lewy bodies, which represent a neuropathological hallmark of PD. We observed that CeO_2_ NPs strongly reduce α-syn-induced toxicity in a dose-dependent manner. This effect is associated with the inhibition of cytoplasmic α-syn foci accumulation, resulting in plasma membrane localization of α-syn after NP treatment. Moreover, CeO_2_ NPs counteract the α-syn-induced mitochondrial dysfunction and decrease reactive oxygen species (ROS) production in yeast cells. In vitro binding assay using cell lysates showed that α-syn is adsorbed on the surface of CeO_2_ NPs, suggesting that these NPs may act as a strong inhibitor of α-syn toxicity not only acting as a radical scavenger, but through a direct interaction with α-syn in vivo.

## 1. Introduction

Neurodegenerative proteinopathies are characterized by the formation of misfolded protein aggregates, which cause cellular toxicity and contribute to cellular proteostatic collapse [1]. The human α-synuclein (α-syn), a small lipid-binding protein localized primarily to presynaptic terminals, represents the major component of Lewy bodies, insoluble cytoplasmic aggregates found in the neurons of Parkinson’s disease (PD) patients [2]. Although the exact function of α-syn remains unknown, it seems to be involved in the regulation of vesicle trafficking network at the synapses of dopaminergic neurons [3,4] and its dysfunction appears to be a critical determinant for the development of PD [5,6]. Genetic mutations, as well as duplications or triplications of the α-syn locus, are indeed identified in familial forms of PD [7,8,9], and increased expression of α-syn leads to neurodegeneration in several animal models [10,11].

The structural flexibility of α-syn is critical for its function in vivo [12]. α-Syn is an intrinsically disordered protein with an N-terminal domain that adopts an amphipathic α-helical conformation when associated with membranes [13,14], while the hydrophilic C-terminal domain remains unstructured [15]. In physiological conditions, α-syn exists in a tightly-regulated equilibrium between cytoplasmic monomeric (unstructured) proteins and membrane-bound α-syn with α-helix conformation [13,14,16,17,18]. However, in pathological conditions, α-syn undergo a series of lipid-dependent conformational changes leading to the formation of β-sheet-rich aggregates (α-syn oligomers), considered the most neurotoxic forms of α-syn [19,20,21,22], which can further mature into amyloid fibrils [23]. Molecular mechanisms through which α-syn forms oligomeric aggregates and contributes to neurodegeneration remain unknown, but this phenomenon seems to be associated with an overproduction of α-syn (that would cause an increased local α-syn concentration on membranes [24]), a failure of the proteolytic mechanisms that cleave misfolded α-syn, oxidative stress, and mitochondrial overwork [4,12].

In this scenario, it becomes crucial to identify therapeutic approaches against α-syn pathogenicity. Although lacking the multicellular and physiological complexity of the higher eukaryotes, the yeast *Saccharomyces cerevisiae* is a powerful system for studying the molecular basis of synucleinopathies [6,25,26,27]. Overexpression of human α-syn in yeast cells under the control of a galactose-inducible promoter results in dose-dependent toxicity and global cellular dysfunction [25]. Yeast models of α-syn toxicity recapitulate several salient features of PD [6,25,27,28,29]: vesicle trafficking defects, mitochondrial dysfunction, excessive production of reactive oxygen species (ROS), and impairment of the ubiquitin-proteasome system. Several genetic and chemogenomic screenings conducted using yeast models of PD have identified suppressors of α-syn toxicity which are also effective in neuronal models [27,28,29,30,31,32,33,34,35,36].

Cerium oxide nanoparticles (CeO_2_ NPs) have gained great interest in cancer treatment [37,38,39,40,41,42], protection from ionizing radiation [43,44], prevention of retinal degeneration [45] and neurodegenerative diseases [46,47,48,49,50,51,52]. Together with a high biocompatibility [53,54], CeO_2_ NPs are redox-active materials mimicking enzymes involved in oxidative stress response as superoxide dismutase [55,56] or catalase [57] and, as such, can scavenge ROS and nitric oxide [58]. Depending on the environmental conditions, CeO_2_ NPs can reversibly bind oxygen and Ce can shift between Ce^3+^ and Ce^4+^ on NP surface [59]. The antioxidant properties of these NPs are crucially linked to this Ce^3+^/Ce^4+^ redox switch [58,60,61]. However, CeO_2_ NPs have been also found to display oxidase-like activity at acidic pH [62] and to generate noxious ROS in different organisms and cell systems [63,64,65]. Recently, docking studies revealed that, compared with other nanostructured materials, CeO_2_ NPs best fit in the active site of α-syn and interfere with the formation of fibrillar structures of α-syn formed in vitro [52,66]. Therefore, the aim of the present work was to evaluate the effects of CeO_2_ NPs on α-syn toxicity in a validated yeast model that allows us to investigate whether these NPs affect the formation, accumulation and cellular localization of α-syn in vivo, restoring the molecular pathways altered by α-syn overexpression.

## 2. Materials and Methods

### 2.1. Cerium Oxide and Amorphous Silica Nanoparticles

CeO_2_ NPs (Sigma-Aldrich; <25 nm, particle size) and amorphous silica nanoparticles (ASNPs) used in the present work were previously characterized [50,67,68,69]. ASNPs produced via thermal route (NM-203; 13 nm, mean particle size) were provided by the JRC Nanomaterials Repository (Ispra, Varese, Italy) [69].

Prior to their use, NPs were exposed for 16 min to sonication at room temperature in a Transonic T460/H device (Elma Electronic GmbH, Pforzheim, Germany) to reduce NP aggregation. Zeta potentials and particle size distribution of the NP dispersions were determined by dynamic light scattering (DLS) technique using Zetasizer Nano ZSP (Malvern Instruments Ltd., Malvern, UK). DLS revealed that CeO_2_ NPs present a hydrodynamic diameter of 130 nm in aqueous media, indicating NP aggregation; a zeta potential of 41 mV suggests that CeO_2_ NPs were positively-charged and stable in suspension. ASNPs present a hydrodynamic diameter of 284 nm in aqueous media and a zeta potential of −43 mV.

### 2.2. Yeast Strains and Growth Conditions

A low-efflux mutant (W303 *pdr1Δpdr3Δ*; *MATα can1–100*, *his3–11,15*, *leu2–3,112*, *trp1-1*, *ura3-1*, *ade2-1*, *pdr1::kanMX, pdr3::kanMX*) containing two copies of the gene that encodes the green fluorescent protein (GFP) integrated into the *URA3* and *TRP1* loci was used as reference (wild-type, WT) strain in this work. The HiTox strain (W303 *pdr1Δpdr3Δ* genetic background) carrying two copies of the *α-syn-GFP* gene integrated into the *URA3* and *TRP1* loci was used as PD model. In WT and HiTox strains, the expressions of *GFP* and *α-syn-GFP* genes were under the control of the galactose-inducible *GAL1* promoter. These strains were kindly provided by the laboratory of Susan Lindquist [25,29,32].

WT and HiTox strains were grown at 28 °C in synthetic defined minimal medium containing 0.67% (w/v) yeast nitrogen base without amino acids, adenine (20 μg/mL), histidine (20 μg/mL), leucine (30 μg/mL), and 2% (w/v) glucose (SD medium; ‘repressing’ condition) or galactose (SGal medium; ‘inducing’ condition) as carbon source.

For mitochondrial morphology analysis, WT and HiTox strains were transformed with pYX142 plasmid (*LEU* selectable marker) expressing a mitochondrial-localized red fluorescent protein (mtRFP) provided by the laboratory of Dr. Winderickx (KU Leuven, Leuven, Belgium). Yeast transformants were selected in SD medium lacking leucine.

### 2.3. Cytotoxicity Assays

For serial dilution ‘spot’ assays [70], yeast cells pre-cultured at 28 °C in SD medium for 24 h were collected by centrifugation, washed twice with sterile milliQ water, and adjusted to an OD_600_ (optical density at 600 nm) value of 1.0. Cells were serially diluted in ten-fold increments prior to spotting (4 μL aliquots for each dilution) onto selective SD or SGal agar plates. Yeast growth was examined by visual inspection (and photographically recorded) after incubation at 28 °C for 48 h.

For clonogenic assays [71], yeast pre-cultures in SD medium were collected, washed, diluted to an OD_600_ value of 0.05 with SGal medium and transferred to 96-well plates. Cells were incubated with different concentrations of NPs (10–100 ng/μL) for 48 h at 28 °C without agitation. Parallel controls without NP treatment were also provided. Cultures were then diluted 500-fold with sterile milliQ water and an aliquot of each dilution was seeded in SD agar plates. Colonies were allowed to form for 72 h and colony-forming units (CFU) were recorded.

### 2.4. Enzymatic Cell Wall Degradation

Yeast pre-cultures in SD medium were collected, washed and diluted to an OD_600_ value of 0.2 with SGal medium. Cells were grown for 1.5 h at 28 °C and then treated with lyticase (500 U/OD_600_ unit of cells; Sigma-Aldrich, St. Louis, MO, USA) for 30 min at 28 °C to degrade cell walls. After lyticase treatment, spheroplasts were centrifugated, resuspended in SGal medium and incubated at 28 °C for 4 h in the presence (or not) of CeO_2_ NPs (50 ng/μL). Aliquots of spheroplasts were then analyzed by optical microscopy (see below).

### 2.5. Fluorescence Microscopy Analysis

Image acquisition and analysis were performed with a Zeiss Axio Imager.Z2 fluorescence microscope (Carl Zeiss Microscopy GmbH, Jena, Germany). To ensure reproducibility between experiments, all images were recorded with the same microscope settings.

For the analysis of α-syn-GFP cellular localization, yeast cells were cultivated under the same conditions used for clonogenic assays. Cells with fluorescent cytoplasmic aggregates (foci) or with α-syn associated with plasma membranes were scored by examining 150 cells in at least three independent experiments. The percentage of ‘positive’ cells was calculated relative to the total cell number in the counted fields.

For cell wall staining, yeast cells were collected as previously described, washed in water and stained for 30 min at 28 °C in the dark with calcofluor white M2R (excitation/emission at 365 nm and 435 nm; Thermo Fisher Scientific, Waltham, MA, United States) at a final concentration of 25 μM.

For mitochondrial morphology analysis, yeast cells (starting from an OD_600_ value of 0.1) were grown in SGal medium in the presence or in the absence of CeO_2_ NPs (50 ng/μL) and incubated with agitation for 24 h at 28 °C. Cells were then directly analyzed with a fluorescence microscope (TRITC filter). RFP fluorescence was used to investigate the mitochondrial morphology and to discriminate between two types of mitochondrial morphologies, the wild-type ‘tubular’ and the fragmented mitochondria.

Oxidative stress was detected in yeast cells (starting from an OD_600_ value of 0.1) grown in SGal medium in the presence or in the absence of CeO_2_ NPs (50 ng/µL) and incubated with agitation for 4 h at 28 °C. Cells were then stained for 30 min at 37 °C with CellROX™ Orange Reagent (5 μM, final concentration) according to manufacturer’s instructions (Thermo Fisher Scientific). This cell-permeant dye is non-fluorescent in a reduced state and exhibits bright orange fluorescence (with excitation/emission at 545/565 nm) upon oxidation mediated by ROS generated by various agents.

### 2.6. Flow Cytometry Analysis

Flow cytometry (FC) analysis was conducted using a NovoCyte^®^ flow cytometer (ACEA Biosciences Inc., San Diego, CA, USA) and the images recorded were elaborated using the Novocyte^®^ Express software. At least 20,000 cells (events) were collected in each specimen and analysis was conducted on viable α-syn-expressing cells (FITC positive). For side scatter (SSC) measurement, yeast cells were grown in SGal medium for 24 h and 48 h at 28 °C in the presence of CeO_2_ NPs (50 ng/μL) or ASNPs (100 ng/μL) prior to FC analysis. Parallel controls without NP treatment were also provided.

MitoTracker™ Deep Red (MTDR; Thermo Fisher Scientific), a far red-fluorescent dye (with excitation/emission at 644/665 nm) that stains mitochondria in living cells, was used for mitochondrial labeling analysis with FC. An unstained sample control was used to detect the signal of autofluorescence. Yeast cells were grown in SGal medium for 4 h at 28 °C in the presence (or not) of CeO_2_ NPs (50 ng/μL). Cells were then harvested, resuspended in milliQ water and stained at 28 °C for 30 min with MTDR (at a final concentration of 0.5 nM) according to the manufacturer’s instructions (Thermo Fisher Scientific).

### 2.7. Protein Extractions and NP-Protein Binding Assay

Cell extracts were prepared from yeast cultures grown in SGal medium for 6 h at 28 °C in the presence (or not) of CeO_2_ NPs (25–50 ng/μL). Cells were harvested by centrifugation, resuspended in ice-cold lysis buffer (25 mM Tris-HCl pH 7.5, 50 mM KCl, 1 mM MgCl_2_, 1 mM EDTA, 1% Triton X-100, 10% glycerol, and protease inhibitors) supplemented with an equal volume of glass beads (0.5 mm diameter), and lysed by five rounds of vigorous vortexing (1 min stroke followed by 5 min incubation on ice) with a Mini-Beadbeater-16 (BioSpec Products Inc., Bartlesville, OK, USA). The resulting lysates were clarified by centrifugation (at 14,000 rpm for 30 min, 4 °C) and the protein concentration in each supernatant fractions was determined with the Bradford reagent (Bio-Rad, Hercules, CA, USA).

Yeast protein extract obtained from untreated HiTox strain (800 μg total protein) was used for α-syn binding in vitro using an experimental protocol described previously [72]. Protein extract from WT strain (which does not express α-syn) was used as a negative control. Briefly, cell extracts (7 g/L protein) were incubated in the presence of 0.5 g/L CeO_2_ NPs in phosphate-buffered saline (PBS) at 4 °C for 16 h with gentle agitation. CeO_2_ NPs, along with their adsorbed yeast proteins, were recovered by ultracentrifugation (at 40,000 rpm for 20 min, 4 °C), and unbound proteins were removed by rinsing the pellet five times in PBS. After each rinse, the pellets were gently vortexed, re-centrifuged (at 40,000 rpm for 20 min, 4 °C) and supernatants were discarded. Adsorbed proteins were eluted from the NP surface by a 1 h incubation at room temperature in PBS, then denatured at 100 °C for 5 min.

### 2.8. Dot Blot Analysis

Dot blot analysis was performed in a 96-well plate format using a Bio-Dot^®^ microfiltration apparatus (Bio-Rad, Herakles, CA, USA) for vacuum-transfer of the samples to nitrocellulose membranes (0.2 μm pore-size; Bio-Rad) pre-wetted with Tris-buffered saline (TBS; 20 mM Tris-HCl pH 7.5, 0.8% NaCl). Sample-loaded membranes were then blocked by incubation for 2 h at room temperature in TTBS (TBS supplemented with 0.1% Tween 20) containing 5% (w/v) bovine serum albumin (BSA). After blocking, membranes were incubated overnight at 4 °C with an anti-α-syn antibody (Santa Cruz Biotechnology Inc., Dallas, TX, USA; 1:200 dilution). An anti-Pgk1 antibody (Abcam, Cambridge, UK; 1:2000 dilution) was used as a loading control. Following washing with TTBS, membranes were incubated for 1 h at room temperature with IRDye-labeled goat anti-rabbit (for anti-α-syn) or goat anti-mouse (for anti-Pgk1) secondary antibodies (LI-COR Biosciences, Lincoln, NE, United States; 1:10000 dilution). Following washing with TTBS, membranes were dried and visualized with a Chemidoc MP Imaging System (Bio-Rad).

### 2.9. Statistical Analysis

Statistical analysis was performed using GraphPad Prism v6.0. For each experiment, three biological replicates and at least three technical replicates were performed. Statistical analysis was performed using one-way ANOVA, followed by Dunnett’s multiple comparisons test (*, *p* < 0.05; **, *p* < 0.01; ***, *p* < 0.001; ****, *p* < 0.0001).

## 3. Results

### 3.1. CeO_2_ NPs Counteract α-syn-induced Toxicity in the Yeast Model System

**Figure 1:**
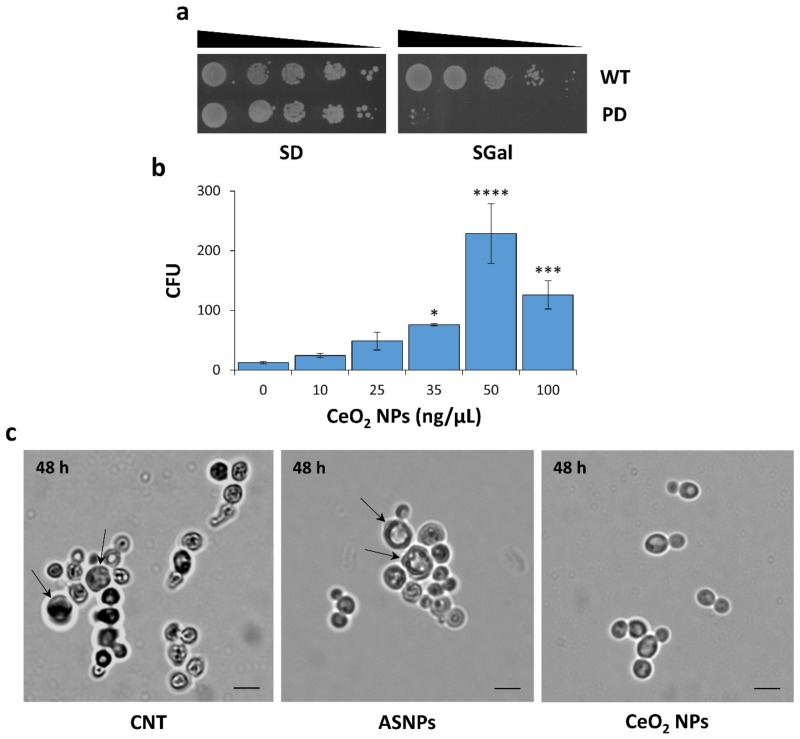
CeO_2_ NPs strongly increase the viability of yeast cells expressing human α-syn. (**a**) Serial dilution spot assays (10-fold input cell dilutions ranging from 10^1^ to 10^4^) were performed on wild-type (WT) and HiTox (PD) strains. Cells were spotted on agar plates containing glucose (SD; ‘repressing’ conditions) or galactose (SGal; ‘inducing’ conditions) as a carbon source, and growth was assessed after 48 h at 28 °C. The extreme toxicity of α-syn expressed in the PD model under the control of the galactose-inducible promoter is shown in the *right* panel. (**b**) Dose-response plot of the protective effects of CeO_2_ NPs against α-syn toxicity determined by clonogenic assay. The number of colony-forming units (*CFU*) developed on agar plates at 28 °C for 72 h were recorded and counted manually. Data are the mean ± SD of three independent experiments performed at least in triplicate. Significance was determined by one-way ANOVA with Dunnett’s multiple comparisons test. *, *p* < 0.05; ***, *p* < 0.001; ****, *p* < 0.0001. (**c**) α-Syn-expressing cells were grown with or without ASNPs (50 ng/μL) or CeO_2_ NPs (50 ng/μL) for 48 h at 28 °C prior to observation. Large mother cells are indicated with black arrows. Phase-contrast microscopy images were shown. *CNT* indicates the control (untreated) sample, and scale bars were set at 5 μm.

In the present study, we used a validated yeast model of PD (HiTox strain) [25,26,28,30] to investigate the effects of CeO_2_ NPs on α-syn-induced toxicity. This strain contains two copies of the gene encoding α-syn fused to GFP integrated into the yeast genome under the control of a galactose-inducible promoter. In ‘inducing’ growth conditions (SGal medium), α-syn is overexpressed and causes a strong inhibition of the yeast cell growth compared with the reference strain (Figure 1a). A clonogenic assay was performed using HiTox strain (see ‘Materials and Methods’ for details) to assess whether CeO_2_ NPs can counteract the α-syn-induced toxicity. Yeast cells were grown at 28 °C in selective SGal medium and exposed to different concentrations of CeO_2_ NPs (10–100 ng/μL) for 48 h. Aliquots of each sample were then diluted and seeded in selective glucose-containing (SD) agar plates. The number of CFU was recorded after 72 h of incubation at 28 °C in control (untreated) and treated samples. As shown in Figure 1b, CeO_2_ NPs strongly increased the viability of the α-syn-expressing cells, when compared with control samples. Clonogenic assay displayed a bell-shaped dose-response curve (Figure 1b), showing that the beneficial effect of CeO_2_ NPs was attenuated at the highest concentration tested (100 ng/μL).

To evaluate whether the effect observed was specific for these NPs, the clonogenic assay was also performed in the presence of ASNPs (10-100 ng/μL), another type of biocompatible NPs that seem to interact with α-syn [73]. In contrast with CeO_2_ NPs, ASNPs did not counteract the toxicity of α-syn in yeast cells, but at the highest dose (100 ng/µL) further reduced the viability of the HiTox strain (50% reduction of viability compared with the untreated sample).

After 48 h of growth in ‘inducing’ conditions, yeast cells were monitored using an optical microscope (Figure 1c). As expected, untreated cells or cells treated with ASNPs showed an altered and heterogeneous morphology resembling the senescence-like phenotype reported for aged cells with the presence of bigger mother cells and smaller round-shaped daughters [74,75]. Conversely, oval-shaped budding cells were observed after the treatment with CeO_2_ NPs, indicating the presence of dividing normal cells still after 48 h from the induction of α-syn expression.

**Figure 2:**
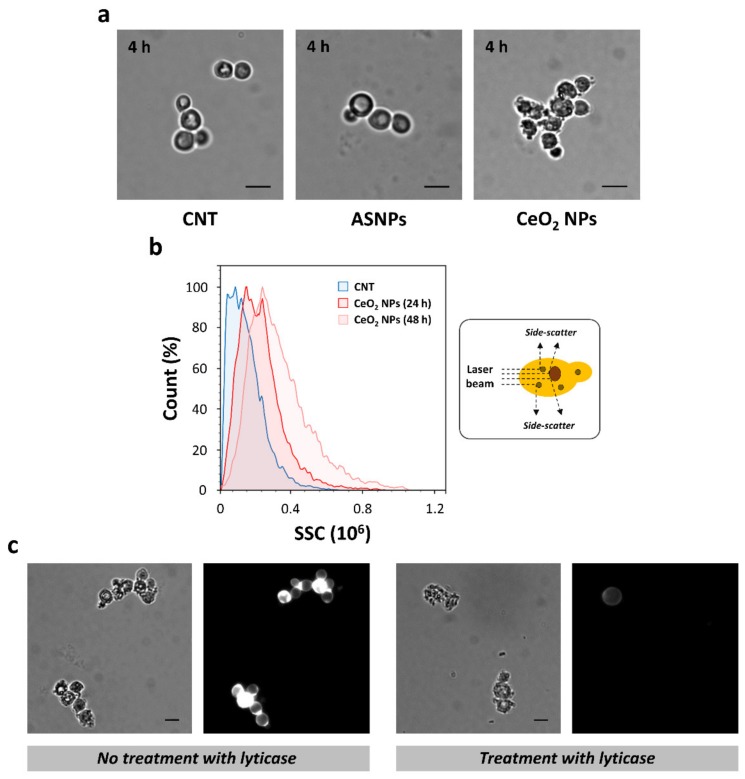
The interaction between CeO_2_ NPs and plasma membranes can promote the beneficial effects of these NPs in yeast. (**a**) α-Syn-expressing cells were grown with or without ASNPs (100 ng/μL) or CeO_2_ NPs (50 ng/μL) for 4 h at 28 °C prior to microscopy observations. *CNT* indicates the control (untreated) sample and scale bars were set at 5 μm. (**b**) Flow cytometry (FC) analysis of α-syn-expressing cells grown on SGal medium with or without CeO_2_ NPs for different times of incubation (24–48 h). A schematic representation of SSC signal detection by FC was illustrated (*right panel*) in which an optical detector measures light scatter at a ninety-degree angle relative to the laser beam of FC. **(c)** Yeast cells untreated (*left*) or treated (*right*) with lyticase were exposed to CeO_2_ NPs (50 ng/μL) for 4 h at 28 °C and then stained with calcofluor white. Phase contrast (*left-side*) and fluorescence (*right-side*) microscopy images were shown. Scale bars were set at 5 μm.

To monitor the effects of CeO_2_ NPs in early phases of growth, we observed yeast cells after a few hours of exposure (4 h) in ‘inducing’ conditions (Figure 2a). Microscope observation revealed that α-syn-expressing yeast cells were ‘caged’ by these NPs, which appeared to be firmly bound to the outside of the cells (Figure 2a). This effect disappeared after 48 h of growth in the presence of CeO_2_ NPs (Figure 1c) indicating that CeO_2_ NPs can be internalized in a time-dependent manner in yeast cells. To validated this hypothesis, a flow cytometric analysis was then conducted in yeast cells treated (or not) with the most effective concentration of CeO_2_ NPs (50 ng/μL). Using a gating strategy that eliminated dead cells, cellular debris and smaller-sized particulates, we analyzed viable α-syn-expressing cells to generate histogram plots for side scatter (SSC) signals (Figure 2b). SSC data provide information about the internal complexity (i.e., granularity) of the cell, and previous reports have shown a correlation between increasing SSC signals and cellular uptake of NPs [76,77,78,79,80]. A time-dependent increase of SSC signals was observed in CeO_2_ NP-treated cells compared with control cells, indicating that these NPs were internalized in yeast cells (Figure 2b).

To assess whether components of the cell wall or plasma membrane can mediate the adsorption of CeO_2_ NPs, yeast cells were pretreated with lyticase, a hydrolytic enzyme that selectively degrades the cell wall of vegetative cells. Cell wall digestion was assessed by staining with calcofluor white, a chitin/chitosan-binding dye (Figure 2c). Cells pretreated with lyticase showed degraded cell walls and a high adsorption of CeO_2_ NPs on the cell surface (Figure 2c). The interaction between CeO_2_ NPs and plasma membranes could represent an important step in the cellular uptake of these NPs. Considering the zeta potential of CeO_2_ NPs (41 mV; see ‘Materials and Methods’ for details), it is possible that an electrostatic interaction with negatively-charged plasma membranes can favour the adsorption of these NPs. Of note, no interaction with the cell surface was observed with negatively-charged ASNPs (zeta potential, −43 mV) even at the highest concentration tested (100 ng/µL; Figure 2a). ASNPs appeared to be internalized in yeast to a lesser extent respect to CeO_2_ NPs (Figure S1) and a different mechanism may be responsible for the uptake of these negatively-charged NPs [81].

### 3.2. CeO_2_ NPs Counteract α-syn Foci Formation

**Figure 3:**
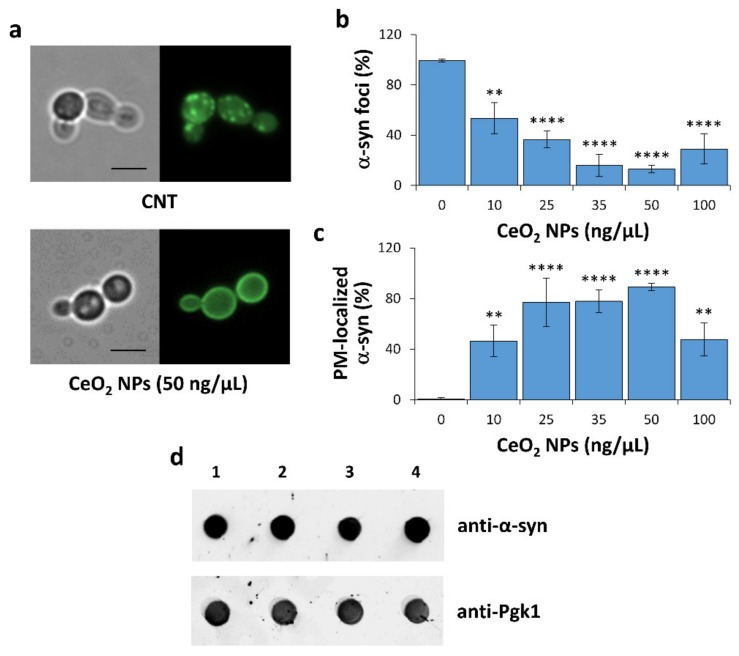
CeO_2_ NPs affect α-syn localization and toxic foci accumulation. (**a**) Microscope observation of α-syn-GFP-expressing cells (HiTox strain) grown with or without CeO_2_ NPs (50 ng/µL) for 48 h at 28 °C. For each sample, phase contrast (*left*) and fluorescence (*right*) microscopy images were shown. *CNT* indicates the control (untreated) sample, and scale bars were shown at 5 µm. (**b**) Yeast cells were treated with different concentrations of CeO_2_ NPs (10–100 ng/µL) for 48 h at 28 °C and the percentages of cells with α-syn cytoplasmic foci were quantified relative to the total number of cells. Significance was determined by one-way ANOVA with Dunnett’s multiple comparisons test (**, *p* < 0.01; ****, *p* < 0.0001). (**c**) Yeast cells were treated as indicated in (**b**) and the percentages of cells with α-syn associated to the plasma membrane (PM) were quantified relative to the total number of cells. (**d**) Representative results of a dot blot analysis performed with an anti-α-syn antibody on whole cell extract samples (10 µg total protein each) derived from untreated (1) or NP-treated samples [CeO_2_ NP concentrations: 25 ng/µL (2); 35 ng/µL (3); 50 ng/µL (4)]. Immunoreactivity with the constitutively expressed phoshoglycerate kinase enzyme (Pgk1) served as a loading control.

When expressed in yeast, α-syn first accumulates at the plasma membrane in a highly selective manner before forming cytoplasmic inclusions (intracellular foci; Figure 3a, upper panel) that cause cellular toxicity [25,27,28,30]. Interestingly, the formation of α-syn foci and the resulting growth defect were reversed by genetic and chemical suppressors of α-syn toxicity that are also effective in human cells and animal models [27,28,29,30,31,32,34,35,36].

The effects of CeO_2_ NPs on the formation, accumulation and localization of α-syn–GFP cytoplasmic aggregates were therefore analyzed (Figure 3). Treatment with CeO_2_ NPs strongly reduced the formation of fluorescent cytoplasmic inclusions of α-syn in a dose-dependent manner (Figure 3a,b) and this effect correlated with the efficacy of CeO_2_ NPs to counteract α-syn toxicity (Figure 1b). In addition, exposure to CeO_2_ NPs causes a nearly exclusive plasma membrane localization of α-syn (Figure 3a,c).

To exclude the possibility that CeO_2_ NPs do not simply inhibit the expression of α-syn driven by *GAL1* promoter, a dot blot analysis using an anti-α-syn antibody was performed to evaluate the expression levels of this protein (Figure 3d). An anti-Pgk1 antibody was used as housekeeping/loading control. No measurable reduction for α-syn abundance was observed upon NP treatment compared with untreated sample (Figure 3d). These results suggest a possible direct interaction of these NPs with α-syn in monomeric or oligomeric state.

### 3.3. CeO_2_ NPs Reverse Mitochondrial Damage and Decrease ROS Production Induced by α-syn Overexpression

Mitochondrial stress is an early signature of α-syn toxicity in both yeast and mammalian cells [32,82,83]. To analyze mitochondrial morphology in living cells, HiTox strain was transformed with a plasmid expressing a mitochondrial-localized red fluorescent protein (mtRFP). The import of mtRFP into the mitochondrial compartment appears not to be hampered in respiratory-deficient mutants with reduced membrane potential. The possibility of a direct microscope observation without the need of pre-incubation, the relative independence on the mitochondrial membrane potential, and the relatively stable fluorescence of RFP are obvious advantages of the use of mtRFP for mitochondrial morphology analysis.

**Figure 4:**
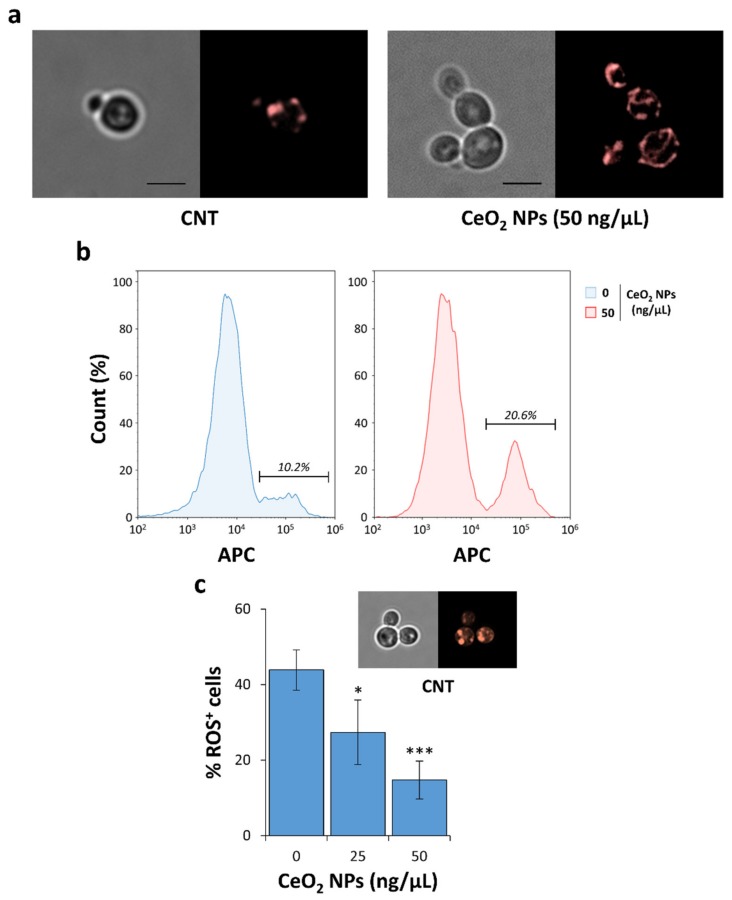
CeO_2_ NPs restore mitochondrial morphology and decrease ROS production in α-syn-expressing cells. (**a**) HiTox strain transformed with a construct for the expression of mitochondrial-localized RFP was grown in ‘inducing’ medium (SGal) for 24 h with (*right*) or without (*left*) CeO_2_ NPs (50 ng/µL). For each sample, phase contrast (*left-side*) and fluorescence (*right-side*) microscopy images were shown. *CNT* indicates the control (untreated) sample, and scale bars were set at 5 µm. (**b**) Yeast cells were grown in SGal medium for 4 h with or without CeO_2_ NPs (50 ng/µL). Active mitochondria in α-syn-expressing (GFP-positive) cells were monitored by MitoTracker Deep Red (MTDR) fluorescence using flow cytometer analysis in APC channel. Representative histogram plots were shown, and the percentages of MTDR-positive mitochondria in α-syn-expressing cells were indicated for each sample. (**c**) After NP exposure for 4 h, the cells were stained with CellROX^®^ Orange Reagent and visualized with fluorescence microscopy. CellROX-positive cells were scored. A representative microscope image is shown above the graph. Significance was determined using one-way ANOVA with Dunnett’s multiple comparisons test (*, *p* < 0.05; ***, *p* < 0.001).

In wild-type yeast cells, mitochondria form an extensive tubular reticulum, and this morphology is maintained by coordinated fission and fusion events. This network disintegrates during stress conditions that cause cell toxicity, yielding smaller mitochondria [84,85,86]. We observed that, after 24 h of induction, α-syn-expressing cells showed fluorescent punctuated structures indicating the presence of fragmented mitochondria (Figure 4a, left panel). Conversely, the treatment with CeO_2_ NPs at the most effective concentration (50 ng/µL; Figure 1b) restored the wild-type mitochondrial morphology (Figure 4a, right panel) consisting in a branched tubular network. These results underlined how these NPs can prevent not only the cytotoxicity, but also the mitochondrial fragmentation induced by α-syn overexpression.

Protective effects of CeO_2_ NPs on mitochondrial functionality were also tested after few hours (4 h) from the induction of α-syn expression in yeast cells. Flow cytometric analyses were conducted using MTDR, a specific dye that selectively stains undamaged mitochondria in living cells. MTDR passively diffuses across the plasma membrane and then accumulates in active mitochondria in a potential-dependent manner, and subsequently, it covalently binds the thiol groups of the cysteine residues of mitochondrial proteins [87]. Using a gating strategy, we showed that α-syn-expressing cells had a significantly higher amount of actively respiring mitochondria (50% increment) already after a few hours of NP treatment (Figure 4b). Overall, these results confirmed that CeO_2_ NP exposure improves mitochondrial functionality in HiTox strain.

Mitochondrial damage determined by an overexpression of α-syn in yeast cells causes a massive generation of ROS [32,88]. To quantify the generation of ROS in living cells, we used a dye (CellROX) which allows to detect several types of ROS in vivo. As already observed in an Alzheimer’s disease (AD) mouse model [49], CeO_2_ NPs significantly decreased the free radical pool in the yeast PD model (Figure 4c).

### 3.4. CeO_2_ NPs Directly Interact with α-syn In Vitro

**Figure 5:**
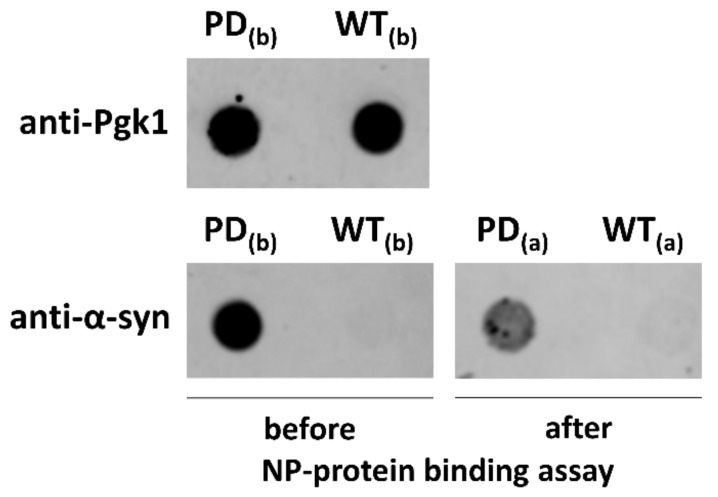
α-Syn is adsorbed on the surface of CeO_2_ NPs. Yeast protein extracts obtained from HiTox (PD) and WT strains were used for an in vitro NP-protein binding assay. Adsorbed proteins from HiTox and WT strains (*PD_(a)_* and *WT_(a)_*, respectively) were recovered from CeO_2_ NP surface by a centrifugation-washing procedure and analyzed by dot blot using an anti-α-syn antibody. Aliquots (5 µg) of HiTox and WT protein extracts (*PD_(b)_* and *WT_(b)_*, respectively) were used as controls for immunodetection. An anti-Pgk1 antibody was used as loading control for protein extracts. *PD_(b)_*, protein extract from the HiTox strain (5 µg); *WT_(b)_*, protein extract from the WT strain (5 µg); *PD_(a)_*, absorbed proteins from an in vitro binding assay performed with protein extracts from the HiTox strain; *WT_(a)_*, absorbed proteins from an in vitro binding assay performed with protein extracts from the WT strain.

High-throughput chemical screenings show that the action of compounds that strongly counteract α-syn toxicity in yeast do not seem to be strictly related to their antioxidant activity, although these compounds cause a marked reduction of ROS generation in yeast cells [32]. It is then possible that the beneficial effects observed with the treatment of CeO_2_ NPs may be mainly due to a direct action of these NPs on α-syn aggregate formation and that a reduction of mitochondrial damage and ROS generation may be related to a reduced formation of α-syn toxic aggregates mediated by NPs. To validate this hypothesis, yeast proteins adsorbed on the NP surface were isolated using a previously developed protocol for the identification of hard corona proteins [72]. Protein extracts obtained from WT and HiTox strains were incubated with gentle agitation in the presence of CeO_2_ NPs. After incubation for 24 h, the NPs and the absorbed proteins were recovered by centrifugation (see ‘Materials and Methods’ for details). Unbound proteins were washed out, and yeast proteins adsorbed with high-affinity were eluted out from the NP surface and then analyzed using dot blot analysis with an anti-α-syn antibody (Figure 5). Dot blot analysis showed the presence of α-syn in the fractions adsorbed to the CeO_2_ NP surface, indicating a direct interaction between CeO_2_ NPs and α-syn. These results agree with recently published papers which show how CeO_2_ NPs have the best fitting for the active site of α-syn using a computational approach [52,66].

## 4. Discussion

In this study, we investigated the effect of the CeO_2_ NPs on α-syn-mediated toxicity using a yeast model of PD (HiTox strain) which has been successfully used not only for high-throughput chemical and genetic screenings, but also to study molecular mechanisms of PD [25,89,90]. In this model, overexpression of α-syn perturbs vesicle trafficking in yeast and causes inhibition of cell growth in association with the formation of cytoplasmic inclusions formed by clusters of vesicles that contain monomeric or oligomeric aggregates of α-syn, but not amyloid fibrils [29,89,91]. This is in agreement with the results recently reported by Shahmoradian and colleagues [92] which show that the Lewy bodies in PD patients consist of lipid vesicle clusters and fragmented organelles, including mitochondria, with high local concentrations of non-fibrillar α-syn molecules. An abnormal formation of vesicle clusters mediated by α-syn in pathological conditions could cause a failure to achieve the correct distributions of these vesicles [18]. Interactions with phospholipid membrane surface are then linked not only to the biological function of α-syn but also to its role in the etiology of PD. α-Syn can misfold, aggregate and form fibrils when it is in contact with phospholipid vesicles [28,33,93,94,95,96]. Moreover, all genetic mutations so far identified in the α-syn (*SNCA*) gene which leads to familial PD are located in the N-terminal lipid-binding domain of this protein, and mutations in components that affect vesicle trafficking are important risk factors for PD [97].

Increasing interest for nanostructured materials [98] and, in particular, to CeO_2_ NPs in neurodegenerative diseases is due to their properties as ROS scavengers and to the small size of these NPs that allows their passage through the blood–brain barrier (BBB) [41,99,100,101]. CeO_2_ NPs protect neurons from ROS-induced damage [102] and alleviate clinical symptoms in different models of neurodegenerative diseases, as AD [47,103] or multiple sclerosis [99]. These neuroprotective effects have been attributed to the prevention of ROS generation, reduction of mitochondrial fragmentation, attenuation of apoptosis, and regulation of signal transduction pathways involved in neuroprotection mediated by CeO_2_ NPs [47,99,103]. So far, few reports have focused on the treatment of PD models with CeO_2_ NPs. CeO_2_ NPs preserve neuronal viability on a 1-methyl-4-phenyl-1,2,3,6-tetrahydropiridine (MPTP)-mouse model of PD [104] and improve motor performance in a 6-hydroxydopamine (OHDA)-induced mouse model of PD [50].

Here, we show that CeO_2_ NPs markedly reduce α-syn cytotoxicity in a dose-dependent manner and affect the cellular localization of α-syn, resulting in a nearly exclusive plasma membrane-associated localization, as observed upon treatment with other bioactive compounds [27,32]. In yeast, α-syn associates early with the plasma membrane, and transitions from the plasma membrane to foci of stalled vesicles was observed when it accumulates to toxic levels [27,32]. CeO_2_ NPs drastically inhibit the accumulation in vivo of α-syn cytoplasmic aggregates and restore molecular pathways altered by α-syn overexpression, without lowering the expression of this protein. As observed in an AD-mouse model [47], CeO_2_ NPs prevent mitochondrial fragmentation and ROS overproduction. These effects are more probably due to a direct interaction with α-syn, which prevents the formation of its toxic aggregates, rather than to the antioxidant properties of these NPs. Moreover, in vitro binding assays with yeast protein extracts show that α-syn is adsorbed with high affinity onto the surface of CeO_2_ NPs.

In addition, our data suggest that CeO_2_ NPs are time-dependently internalized by yeast cells and that a fast (and strong) interaction with the plasma membranes may positively influence this process. Consistently, it is known that CeO_2_ NPs are internalized by neurons and accumulate in biological membranes of mammalian cells [47,105]. Although the mechanism for cellular uptake of CeO_2_ NPs needs further study in yeast, we can speculate that these NPs can be internalized with a two-step process. The attachment of the NPs to the plasma membrane can represent the first crucial step mediated by electrostatic interactions with negatively-charged phospholipid headgroups. Subsequently, NPs can be internalized via several mechanisms like pinocytosis or endocytosis. The adsorption via electrostatic interaction could also lead to a bending of the membrane, favoring endocytosis for cellular uptake.

It is also possible that the strong binding capacity of these positively-charged NPs to the plasma membranes could also promote the physical interaction between NPs and α-syn. We have in fact observed that negatively-charged ASNPs, which do not show any detectable interactions with the plasma membranes in yeast cells, do not have protective effects on the growth of the PD strain and could even contribute to the aggregation (and then to the toxicity) of α-syn, as observed in mammalian cells [106] and for other NPs [107,108].

When α-syn is bound to vesicles, the highly aggregation-prone non-amyloid component (NAC) region (residues 61–95) of this protein is in an α-helical conformation and acts as a membrane sensor [109]. Since the N-terminal domain and NAC region are necessary for initiating the α-syn misfolding, small molecules (as CeO_2_ NPs) that are capable of stabilizing the physiological α-helical vesicle-bound conformation of α-syn might interfere with its pathogenic aggregation [13,94]. Docking studies [52,66] showed that CeO_2_ NPs exhibit excellent interactions with crucial residues of the N-terminal membrane-bound domain of α-syn, and it is possible that the interaction with this domain can prevent the conformational changes leading to misfolding and aggregation of α-syn. Further studies are required to better elucidate this issue, but the present work shows how the use of a simplified model of PD can be informative to assess the anti-aggregation properties of small molecules and better understand how α-syn aggregates can acquire their neurotoxic properties and interact with metabolic pathways. Evolutionarily-conserved key molecular components shared between yeast and neurons make it a good model for the study of cellular metabolic events involved in PD [110], but considering the highly specialized function of neurons, yeast studies represent only the starting point to understand such complex diseases. The distance of this study conducted in a yeast model system from an application to a novel treatment of PD is still far; nevertheless, the knowledge obtained in this work may open new possibilities to target PD and related synucleinopathies.

## Supplementary Materials

The following are available online at https://www.mdpi.com/2079-4991/10/2/235/s1, Figure S1: Flow cytometry analysis of α-syn-expressing cells exposed to ASNPs.

## References

[1] Sweeney P., Park H., Baumann M., Dunlop J., Frydman J., Kopito R., McCampbell A., Leblanc G., Venkateswaran A., Nurmi A. (2017). Protein misfolding in neurodegenerative diseases: Implications and strategies. Transl. Neurodegener..

[2] Spillantini M.G., Schmidt M.L., Lee V.M., Trojanowski J.Q., Jakes R., Goedert M. (1997). Alpha-synuclein in Lewy bodies. Nature.

[3] Tofaris G.K., Spillantini M.G. (2007). Physiological and pathological properties of alpha-synuclein. Cell. Mol. Life Sci..

[4] Lashuel H.A., Overk C.R., Oueslati A., Masliah E. (2013). The many faces of alpha-synuclein: From structure and toxicity to therapeutic target. Nat. Rev. Neurosci..

[5] Lee V.M., Trojanowski J.Q. (2006). Mechanisms of Parkinson’s disease linked to pathological alpha-synuclein: New targets for drug discovery. Neuron.

[6] Auluck P.K., Caraveo G., Lindquist S. (2010). alpha-Synuclein: Membrane interactions and toxicity in Parkinson’s disease. Annu. Rev. Cell Dev. Biol..

[7] Polymeropoulos M.H., Lavedan C., Leroy E., Ide S.E., Dehejia A., Dutra A., Pike B., Root H., Rubenstein J., Boyer R. (1997). Mutation in the alpha-synuclein gene identified in families with Parkinson’s disease. Science.

[8] Chartier-Harlin M.C., Kachergus J., Roumier C., Mouroux V., Douay X., Lincoln S., Levecque C., Larvor L., Andrieux J., Hulihan M. (2004). Alpha-synuclein locus duplication as a cause of familial Parkinson’s disease. Lancet.

[9] Singleton A.B., Farrer M., Johnson J., Singleton A., Hague S., Kachergus J., Hulihan M., Peuralinna T., Dutra A., Nussbaum R. (2003). alpha-Synuclein locus triplication causes Parkinson’s disease. Science.

[10] Feany M.B., Bender W.W. (2000). A Drosophila model of Parkinson’s disease. Nature.

[11] Lakso M., Vartiainen S., Moilanen A.M., Sirvio J., Thomas J.H., Nass R., Blakely R.D., Wong G. (2003). Dopaminergic neuronal loss and motor deficits in Caenorhabditis elegans overexpressing human alpha-synuclein. J. Neurochem..

[12] Ghiglieri V., Calabrese V., Calabresi P. (2018). Alpha-Synuclein: From Early Synaptic Dysfunction to Neurodegeneration. Front. Neurol..

[13] Bartels T., Choi J.G., Selkoe D.J. (2011). alpha-Synuclein occurs physiologically as a helically folded tetramer that resists aggregation. Nature.

[14] Killinger B.A., Melki R., Brundin P., Kordower J.H. (2019). Endogenous alpha-synuclein monomers, oligomers and resulting pathology: Let’s talk about the lipids in the room. NPJ Parkinsons Dis..

[15] Huang M., Wang B., Li X., Fu C., Wang C., Kang X. (2019). alpha-Synuclein: A Multifunctional Player in Exocytosis, Endocytosis, and Vesicle Recycling. Front. Neurosci..

[16] McLean P.J., Kawamata H., Ribich S., Hyman B.T. (2000). Membrane association and protein conformation of alpha-synuclein in intact neurons. Effect of Parkinson’s disease-linked mutations. J. Biol. Chem..

[17] Perrin R.J., Woods W.S., Clayton D.F., George J.M. (2000). Interaction of human alpha-Synuclein and Parkinson’s disease variants with phospholipids. Structural analysis using site-directed mutagenesis. J. Biol. Chem..

[18] Dettmer U., Ramalingam N., von Saucken V.E., Kim T.E., Newman A.J., Terry-Kantor E., Nuber S., Ericsson M., Fanning S., Bartels T. (2017). Loss of native alpha-synuclein multimerization by strategically mutating its amphipathic helix causes abnormal vesicle interactions in neuronal cells. Hum. Mol. Genet..

[19] Karpinar D.P., Balija M.B., Kugler S., Opazo F., Rezaei-Ghaleh N., Wender N., Kim H.Y., Taschenberger G., Falkenburger B.H., Heise H. (2009). Pre-fibrillar alpha-synuclein variants with impaired beta-structure increase neurotoxicity in Parkinson’s disease models. EMBO J..

[20] Winner B., Jappelli R., Maji S.K., Desplats P.A., Boyer L., Aigner S., Hetzer C., Loher T., Vilar M., Campioni S. (2011). In vivo demonstration that alpha-synuclein oligomers are toxic. Proc. Natl. Acad. Sci. USA.

[21] Ingelsson M. (2016). Alpha-Synuclein Oligomers-Neurotoxic Molecules in Parkinson’s Disease and Other Lewy Body Disorders. Front. Neurosci..

[22] Fusco G., Chen S.W., Williamson P.T.F., Cascella R., Perni M., Jarvis J.A., Cecchi C., Vendruscolo M., Chiti F., Cremades N. (2017). Structural basis of membrane disruption and cellular toxicity by alpha-synuclein oligomers. Science.

[23] Araki K., Yagi N., Aoyama K., Choong C.J., Hayakawa H., Fujimura H., Nagai Y., Goto Y., Mochizuki H. (2019). Parkinson’s disease is a type of amyloidosis featuring accumulation of amyloid fibrils of alpha-synuclein. Proc. Natl. Acad. Sci. USA.

[24] Galvagnion C., Buell A.K., Meisl G., Michaels T.C., Vendruscolo M., Knowles T.P., Dobson C.M. (2015). Lipid vesicles trigger alpha-synuclein aggregation by stimulating primary nucleation. Nat. Chem. Biol..

[25] Outeiro T.F., Lindquist S. (2003). Yeast cells provide insight into alpha-synuclein biology and pathobiology. Science.

[26] Yeger-Lotem E., Riva L., Su L.J., Gitler A.D., Cashikar A.G., King O.D., Auluck P.K., Geddie M.L., Valastyan J.S., Karger D.R. (2009). Bridging high-throughput genetic and transcriptional data reveals cellular responses to alpha-synuclein toxicity. Nat. Genet..

[27] Tardiff D.F., Tucci M.L., Caldwell K.A., Caldwell G.A., Lindquist S. (2012). Different 8-hydroxyquinolines protect models of TDP-43 protein, alpha-synuclein, and polyglutamine proteotoxicity through distinct mechanisms. J. Biol. Chem..

[28] Cooper A.A., Gitler A.D., Cashikar A., Haynes C.M., Hill K.J., Bhullar B., Liu K., Xu K., Strathearn K.E., Liu F. (2006). Alpha-synuclein blocks ER-Golgi traffic and Rab1 rescues neuron loss in Parkinson’s models. Science.

[29] Gitler A.D., Bevis B.J., Shorter J., Strathearn K.E., Hamamichi S., Su L.J., Caldwell K.A., Caldwell G.A., Rochet J.C., McCaffery J.M. (2008). The Parkinson’s disease protein alpha-synuclein disrupts cellular Rab homeostasis. Proc. Natl. Acad. Sci. USA.

[30] Gitler A.D., Chesi A., Geddie M.L., Strathearn K.E., Hamamichi S., Hill K.J., Caldwell K.A., Caldwell G.A., Cooper A.A., Rochet J.C. (2009). Alpha-synuclein is part of a diverse and highly conserved interaction network that includes PARK9 and manganese toxicity. Nat. Genet..

[31] Kritzer J.A., Hamamichi S., McCaffery J.M., Santagata S., Naumann T.A., Caldwell K.A., Caldwell G.A., Lindquist S. (2009). Rapid selection of cyclic peptides that reduce alpha-synuclein toxicity in yeast and animal models. Nat. Chem. Biol..

[32] Su L.J., Auluck P.K., Outeiro T.F., Yeger-Lotem E., Kritzer J.A., Tardiff D.F., Strathearn K.E., Liu F., Cao S., Hamamichi S. (2010). Compounds from an unbiased chemical screen reverse both ER-to-Golgi trafficking defects and mitochondrial dysfunction in Parkinson’s disease models. Dis. Model. Mech..

[33] Chung C.Y., Khurana V., Auluck P.K., Tardiff D.F., Mazzulli J.R., Soldner F., Baru V., Lou Y., Freyzon Y., Cho S. (2013). Identification and rescue of alpha-synuclein toxicity in Parkinson patient-derived neurons. Science.

[34] Tardiff D.F., Jui N.T., Khurana V., Tambe M.A., Thompson M.L., Chung C.Y., Kamadurai H.B., Kim H.T., Lancaster A.K., Caldwell K.A. (2013). Yeast reveal a ”druggable” Rsp5/Nedd4 network that ameliorates alpha-synuclein toxicity in neurons. Science.

[35] Caraveo G., Auluck P.K., Whitesell L., Chung C.Y., Baru V., Mosharov E.V., Yan X., Ben-Johny M., Soste M., Picotti P. (2014). Calcineurin determines toxic versus beneficial responses to alpha-synuclein. Proc. Natl. Acad. Sci. USA.

[36] Tardiff D.F., Khurana V., Chung C.Y., Lindquist S. (2014). From yeast to patient neurons and back again: Powerful new discovery platform. Mov. Disord..

[37] Asati A., Santra S., Kaittanis C., Perez J.M. (2010). Surface-charge-dependent cell localization and cytotoxicity of cerium oxide nanoparticles. ACS nano.

[38] Wason M.S., Colon J., Das S., Seal S., Turkson J., Zhao J., Baker C.H. (2013). Sensitization of pancreatic cancer cells to radiation by cerium oxide nanoparticle-induced ROS production. Nanomed. Nanotechnol. Biol. Med..

[39] Giri S., Karakoti A., Graham R.P., Maguire J.L., Reilly C.M., Seal S., Rattan R., Shridhar V. (2013). Nanoceria: A rare-earth nanoparticle as a novel anti-angiogenic therapeutic agent in ovarian cancer. PLoS ONE.

[40] Sack M., Alili L., Karaman E., Das S., Gupta A., Seal S., Brenneisen P. (2014). Combination of conventional chemotherapeutics with redox-active cerium oxide nanoparticles--a novel aspect in cancer therapy. Mol. Cancer Ther..

[41] Sack-Zschauer M., Karaman-Aplak E., Wyrich C., Das S., Schubert T., Meyer H., Janiak C., Seal S., Stahl W., Brenneisen P. (2017). Efficacy of Different Compositions of Cerium Oxide Nanoparticles in Tumor-Stroma Interaction. J. Biomed. Nanotechnol..

[42] Corsi F., Caputo F., Traversa E., Ghibelli L. (2018). Not Only Redox: The Multifaceted Activity of Cerium Oxide Nanoparticles in Cancer Prevention and Therapy. Front. Oncol..

[43] Tarnuzzer R.W., Colon J., Patil S., Seal S. (2005). Vacancy engineered ceria nanostructures for protection from radiation-induced cellular damage. Nano Lett..

[44] Colon J., Hsieh N., Ferguson A., Kupelian P., Seal S., Jenkins D.W., Baker C.H. (2010). Cerium oxide nanoparticles protect gastrointestinal epithelium from radiation-induced damage by reduction of reactive oxygen species and upregulation of superoxide dismutase 2. Nanomed. Nanotechnol. Biol. Med..

[45] Chen J., Patil S., Seal S., McGinnis J.F. (2006). Rare earth nanoparticles prevent retinal degeneration induced by intracellular peroxides. Nat. Nanotechnol..

[46] Das M., Patil S., Bhargava N., Kang J.F., Riedel L.M., Seal S., Hickman J.J. (2007). Auto-catalytic ceria nanoparticles offer neuroprotection to adult rat spinal cord neurons. Biomaterials.

[47] Dowding J.M., Song W., Bossy K., Karakoti A., Kumar A., Kim A., Bossy B., Seal S., Ellisman M.H., Perkins G. (2014). Cerium oxide nanoparticles protect against Abeta-induced mitochondrial fragmentation and neuronal cell death. Cell Death Differ..

[48] Eitan E., Hutchison E.R., Greig N.H., Tweedie D., Celik H., Ghosh S., Fishbein K.W., Spencer R.G., Sasaki C.Y., Ghosh P. (2015). Combination therapy with lenalidomide and nanoceria ameliorates CNS autoimmunity. Exp. Neurol..

[49] Kwon H.J., Cha M.Y., Kim D., Kim D.K., Soh M., Shin K., Hyeon T., Mook-Jung I. (2016). Mitochondria-Targeting Ceria Nanoparticles as Antioxidants for Alzheimer’s Disease. ACS Nano.

[50] Hegazy M.A., Maklad H.M., Samy D.M., Abdelmonsif D.A., El Sabaa B.M., Elnozahy F.Y. (2017). Cerium oxide nanoparticles could ameliorate behavioral and neurochemical impairments in 6-hydroxydopamine induced Parkinson’s disease in rats. Neurochem. Int..

[51] Zeng F., Wu Y., Li X., Ge X., Guo Q., Lou X., Cao Z., Hu B., Long N.J., Mao Y. (2018). Custom-Made Ceria Nanoparticles Show a Neuroprotective Effect by Modulating Phenotypic Polarization of the Microglia. Angew. Chem. Int. Ed. Engl..

[52] Zand Z., Khaki P.A., Salihi A., Sharifi M., Qadir Nanakali N.M., Alasady A.A.B., Aziz F.M., Shahpasand M., Hasan A., Falahati M. (2019). Cerium oxide NPs mitigate the amyloid formation of α-synuclein and associated cytotoxicity. Int. J. Nanomed..

[53] Park B., Donaldson K., Duffin R., Tran L., Kelly F., Mudway I., Morin J.P., Guest R., Jenkinson P., Samaras Z. (2008). Hazard and risk assessment of a nanoparticulate cerium oxide-based diesel fuel additive—A case study. Inhal. Toxicol..

[54] O’Brien N., Cummins E. (2010). Ranking initial environmental and human health risk resulting from environmentally relevant nanomaterials. J. Environ. Sci. Health A Tox. Hazard. Subst. Environ. Eng..

[55] Korsvik C., Patil S., Seal S., Self W.T. (2007). Superoxide dismutase mimetic properties exhibited by vacancy engineered ceria nanoparticles. Chem. Commun..

[56] Heckert E.G., Karakoti A.S., Seal S., Self W.T. (2008). The role of cerium redox state in the SOD mimetic activity of nanoceria. Biomaterials.

[57] Pirmohamed T., Dowding J.M., Singh S., Wasserman B., Heckert E., Karakoti A.S., King J.E., Seal S., Self W.T. (2010). Nanoceria exhibit redox state-dependent catalase mimetic activity. Chem. Commun..

[58] Dowding J.M., Dosani T., Kumar A., Seal S., Self W.T. (2012). Cerium oxide nanoparticles scavenge nitric oxide radical (NO). Chem. Commun..

[59] Zhang H., He X., Zhang Z., Zhang P., Li Y., Ma Y., Kuang Y., Zhao Y., Chai Z. (2011). Nano-CeO2 exhibits adverse effects at environmental relevant concentrations. Environ. Sci. Technol..

[60] Celardo I., Pedersen J.Z., Traversa E., Ghibelli L. (2011). Pharmacological potential of cerium oxide nanoparticles. Nanoscale.

[61] Walkey C., Das S., Seal S., Erlichman J., Heckman K., Ghibelli L., Traversa E., McGinnis J.F., Self W.T. (2015). Catalytic Properties and Biomedical Applications of Cerium Oxide Nanoparticles. Environ. Sci. Nano.

[62] Asati A., Santra S., Kaittanis C., Nath S., Perez J.M. (2009). Oxidase-like activity of polymer-coated cerium oxide nanoparticles. Angew. Chem. Int. Ed. Engl..

[63] Cheng G., Guo W., Han L., Chen E., Kong L., Wang L., Ai W., Song N., Li H., Chen H. (2013). Cerium oxide nanoparticles induce cytotoxicity in human hepatoma SMMC-7721 cells via oxidative stress and the activation of MAPK signaling pathways. Toxicol. In Vitro.

[64] Ali D., Alarifi S., Alkahtani S., AlKahtane A.A., Almalik A. (2015). Cerium Oxide Nanoparticles Induce Oxidative Stress and Genotoxicity in Human Skin Melanoma Cells. Cell Biochem. Biophys..

[65] Pulido-Reyes G., Rodea-Palomares I., Das S., Sakthivel T.S., Leganes F., Rosal R., Seal S., Fernandez-Pinas F. (2015). Untangling the biological effects of cerium oxide nanoparticles: The role of surface valence states. Sci. Rep..

[66] Kaushik A.C., Bharadwaj S., Kumar S., Wei D.Q. (2018). Nano-particle mediated inhibition of Parkinson’s disease using computational biology approach. Sci. Rep..

[67] Ciofani G., Genchi G.G., Liakos I., Cappello V., Gemmi M., Athanassiou A., Mazzolai B., Mattoli V. (2013). Effects of cerium oxide nanoparticles on PC12 neuronal-like cells: Proliferation, differentiation, and dopamine secretion. Pharm. Res..

[68] Ciofani G., Genchi G.G., Mazzolai B., Mattoli V. (2014). Transcriptional profile of genes involved in oxidative stress and antioxidant defense in PC12 cells following treatment with cerium oxide nanoparticles. Biochim. Biophys. Acta.

[69] Di Cristo L., Movia D., Bianchi M.G., Allegri M., Mohamed B.M., Bell A.P., Moore C., Pinelli S., Rasmussen K., Riego-Sintes J. (2016). Proinflammatory Effects of Pyrogenic and Precipitated Amorphous Silica Nanoparticles in Innate Immunity Cells. Toxicol. Sci..

[70] Ruotolo R., Marchini G., Ottonello S. (2008). Membrane transporters and protein traffic networks differentially affecting metal tolerance: A genomic phenotyping study in yeast. Genome Biol..

[71] Welch A.Z., Koshland D.E. (2013). A simple colony-formation assay in liquid medium, termed ‘tadpoling’, provides a sensitive measure of Saccharomyces cerevisiae culture viability. Yeast.

[72] Ruotolo R., Pira G., Villani M., Zappettini A., Marmiroli N. (2018). Ring-shaped corona proteins influence the toxicity of engineered nanoparticles to yeast. Environ. Sci. Nano.

[73] Vitali M., Rigamonti V., Natalello A., Colzani B., Avvakumova S., Brocca S., Santambrogio C., Narkiewicz J., Legname G., Colombo M. (2018). Conformational properties of intrinsically disordered proteins bound to the surface of silica nanoparticles. Biochim. Biophys. Acta Gen. Subj..

[74] Lee S.S., Avalos Vizcarra I., Huberts D.H., Lee L.P., Heinemann M. (2012). Whole lifespan microscopic observation of budding yeast aging through a microfluidic dissection platform. Proc. Natl. Acad. Sci. USA.

[75] Kakolyri M., Margaritou A., Tiligada E. (2016). Dimethyl sulphoxide modifies growth and senescence and induces the non-revertible petite phenotype in yeast. FEMS Yeast Res..

[76] Suzuki H., Toyooka T., Ibuki Y. (2007). Simple and easy method to evaluate uptake potential of nanoparticles in mammalian cells using a flow cytometric light scatter analysis. Environ. Sci. Technol..

[77] Zucker R.M., Daniel K.M., Massaro E.J., Karafas S.J., Degn L.L., Boyes W.K. (2013). Detection of silver nanoparticles in cells by flow cytometry using light scatter and far-red fluorescence. Cytom. A.

[78] Toduka Y., Toyooka T., Ibuki Y. (2012). Flow cytometric evaluation of nanoparticles using side-scattered light and reactive oxygen species-mediated fluorescence-correlation with genotoxicity. Environ. Sci. Technol..

[79] Friedrich R.P., Janko C., Poettler M., Tripal P., Zaloga J., Cicha I., Durr S., Nowak J., Odenbach S., Slabu I. (2015). Flow cytometry for intracellular SPION quantification: Specificity and sensitivity in comparison with spectroscopic methods. Int. J. Nanomed..

[80] Njoroge J.M., Yourick J.J., Principato M.A. (2018). A flow cytometric analysis of macrophage- nanoparticle interactions in vitro: Induction of altered Toll-like receptor expression. Int. J. Nanomed..

[81] Foroozandeh P., Aziz A.A. (2018). Insight into Cellular Uptake and Intracellular Trafficking of Nanoparticles. Nanoscale Res. Lett..

[82] Nakamura K., Nemani V.M., Azarbal F., Skibinski G., Levy J.M., Egami K., Munishkina L., Zhang J., Gardner B., Wakabayashi J. (2011). Direct membrane association drives mitochondrial fission by the Parkinson disease-associated protein alpha-synuclein. J. Biol. Chem..

[83] Sampaio-Marques B., Felgueiras C., Silva A., Rodrigues M., Tenreiro S., Franssens V., Reichert A.S., Outeiro T.F., Winderickx J., Ludovico P. (2012). SNCA (alpha-synuclein)-induced toxicity in yeast cells is dependent on sirtuin 2 (Sir2)-mediated mitophagy. Autophagy.

[84] Hoffmann H.P., Avers C.J. (1973). Mitochondrion of yeast: Ultrastructural evidence for one giant, branched organelle per cell. Science.

[85] Shaw J.M., Nunnari J. (2002). Mitochondrial dynamics and division in budding yeast. Trends Cell Biol..

[86] Suen D.F., Norris K.L., Youle R.J. (2008). Mitochondrial dynamics and apoptosis. Genes Dev..

[87] Xiao B., Deng X., Zhou W., Tan E.K. (2016). Flow Cytometry-Based Assessment of Mitophagy Using MitoTracker. Front. Cell. Neurosci..

[88] Büttner S., Bitto A., Ring J., Augsten M., Zabrocki P., Eisenberg T., Jungwirth H., Hutter S., Carmona-Gutierrez D., Kroemer G. (2008). Functional mitochondria are required for alpha-synuclein toxicity in aging yeast. J. Biol. Chem..

[89] Menezes R., Tenreiro S., Macedo D., Santos C.N., Outeiro T.F. (2015). From the baker to the bedside: Yeast models of Parkinson’s disease. Microb. Cell.

[90] Khurana V., Peng J., Chung C.Y., Auluck P.K., Fanning S., Tardiff D.F., Bartels T., Koeva M., Eichhorn S.W., Benyamini H. (2017). Genome-Scale Networks Link Neurodegenerative Disease Genes to alpha-Synuclein through Specific Molecular Pathways. Cell Syst..

[91] Soper J.H., Roy S., Stieber A., Lee E., Wilson R.B., Trojanowski J.Q., Burd C.G., Lee V.M. (2008). Alpha-synuclein-induced aggregation of cytoplasmic vesicles in Saccharomyces cerevisiae. Mol. Biol. Cell.

[92] Shahmoradian S.H., Lewis A.J., Genoud C., Hench J., Moors T.E., Navarro P.P., Castano-Diez D., Schweighauser G., Graff-Meyer A., Goldie K.N. (2019). Lewy pathology in Parkinson’s disease consists of crowded organelles and lipid membranes. Nat. Neurosci..

[93] Perrin R.J., Woods W.S., Clayton D.F., George J.M. (2001). Exposure to long chain polyunsaturated fatty acids triggers rapid multimerization of synucleins. J. Biol. Chem..

[94] Fonseca-Ornelas L., Eisbach S.E., Paulat M., Giller K., Fernandez C.O., Outeiro T.F., Becker S., Zweckstetter M. (2014). Small molecule-mediated stabilization of vesicle-associated helical alpha-synuclein inhibits pathogenic misfolding and aggregation. Nat. Commun..

[95] Jo E., McLaurin J., Yip C.M., St George-Hyslop P., Fraser P.E. (2000). alpha-Synuclein membrane interactions and lipid specificity. J. Biol. Chem..

[96] Fanning S., Haque A., Imberdis T., Baru V., Barrasa M.I., Nuber S., Termine D., Ramalingam N., Ho G.P.H., Noble T. (2019). Lipidomic Analysis of alpha-Synuclein Neurotoxicity Identifies Stearoyl CoA Desaturase as a Target for Parkinson Treatment. Mol. Cell..

[97] Bartels T. (2019). A traffic jam leads to Lewy bodies. Nat. Neurosci..

[98] Rakotoarisoa M., Angelova A. (2018). Amphiphilic Nanocarrier Systems for Curcumin Delivery in Neurodegenerative Disorders. Medicines.

[99] Heckman K.L., DeCoteau W., Estevez A., Reed K.J., Costanzo W., Sanford D., Leiter J.C., Clauss J., Knapp K., Gomez C. (2013). Custom cerium oxide nanoparticles protect against a free radical mediated autoimmune degenerative disease in the brain. ACS Nano.

[100] Gao Y., Chen K., Ma J.L., Gao F. (2014). Cerium oxide nanoparticles in cancer. Onco Targets Ther..

[101] Naz S., Beach J., Heckert B., Tummala T., Pashchenko O., Banerjee T., Santra S. (2017). Cerium oxide nanoparticles: A ‘radical’ approach to neurodegenerative disease treatment. Nanomedicine (Lond).

[102] Schubert D., Dargusch R., Raitano J., Chan S.W. (2006). Cerium and yttrium oxide nanoparticles are neuroprotective. Biochem. Biophys. Res. Commun..

[103] D’Angelo B., Santucci S., Benedetti E., Di Loreto S., Phani R.A., Falone S., Amicarelli F., Ceru M.P., Cimini A. (2009). Cerium oxide nanoparticles trigger neuronal survival in a human Alzheimer disease model by modulating BDNF pathway. Curr. Nanosci..

[104] Dillon C.E., Billings M., Hockey K.S., DeLaGarza L., Rzigalinski B.A. (2011). Cerium oxide nanoparticles protect against MPTP-induced dopaminergic neurodegeneration in a mouse model for Parkinson’s disease. NSTI-Nanotech..

[105] Estevez A.Y., Pritchard S., Harper K., Aston J.W., Lynch A., Lucky J.J., Ludington J.S., Chatani P., Mosenthal W.P., Leiter J.C. (2011). Neuroprotective mechanisms of cerium oxide nanoparticles in a mouse hippocampal brain slice model of ischemia. Free Radic. Biol. Med..

[106] Xie H., Wu J. (2016). Silica nanoparticles induce alpha-synuclein induction and aggregation in PC12-cells. Chem. Biol. Interact..

[107] Alvarez Y.D., Fauerbach J.A., Pellegrotti J.V., Jovin T.M., Jares-Erijman E.A., Stefani F.D. (2013). Influence of gold nanoparticles on the kinetics of alpha-synuclein aggregation. Nano Lett..

[108] Wu J., Xie H. (2016). Effects of titanium dioxide nanoparticles on alpha-synuclein aggregation and the ubiquitin-proteasome system in dopaminergic neurons. Artif. Cells Nanomed. Biotechnol..

[109] Fusco G., De Simone A., Gopinath T., Vostrikov V., Vendruscolo M., Dobson C.M., Veglia G. (2014). Direct observation of the three regions in alpha-synuclein that determine its membrane-bound behaviour. Nat. Commun..

[110] Sarto-Jackson I., Tomaska L. (2016). How to bake a brain: Yeast as a model neuron. Curr. Genet..

