# Supplementary material for: Cerium Oxide Nanoparticles Rescue α-Synuclein-Induced Toxicity in a Yeast Model of Parkinson’s Disease
Source: Nanomaterials (Basel). 2020 Jan 29;10(2):235. doi: 10.3390/nano10020235 (PMC7075201; doi:10.3390/nano10020235)
Supplement: Supplementary file 1 [file nanomaterials-10-00235-s001.pdf]

Supplementary Materials

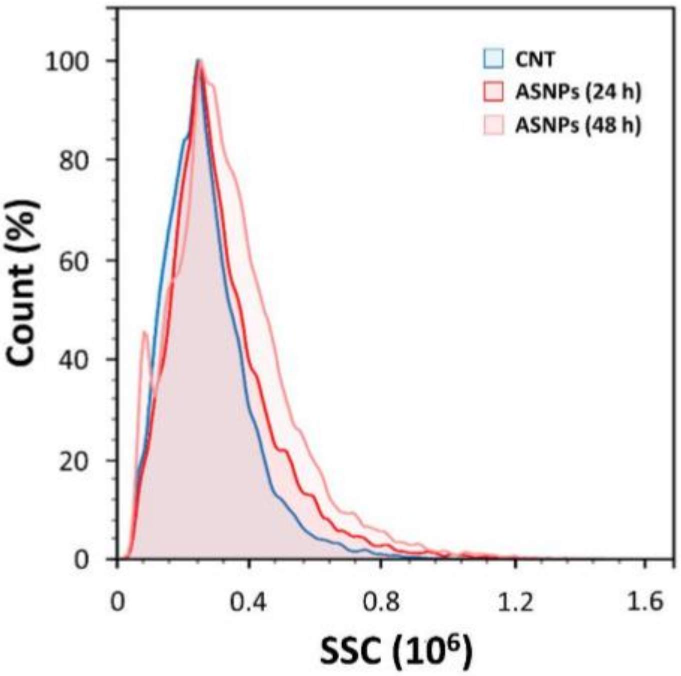

Figure S1. Flow cytometry analysis of α-syn-expressing cells exposed to ASNPs. α-Syn-expressing cells (HiTox strain) were grown on SGal medium with or without ASNPs (100 ng/μL) for different times of incubation (24–48 h) prior to flow cytometry analysis. A time-dependent increase of SSC signals was observed in ASNP-treated cells compared with control (untreated; CNT) cells, indicating that these NPs were internalized in yeast cells, but probably to a lesser extent than CeO_2 NPs (see, for comparison, Figure 2b).
